# The Impact of *PTPRK* and *ROS1* Polymorphisms on the Preeclampsia Risk in Han Chinese Women

**DOI:** 10.1155/2021/3275081

**Published:** 2021-10-04

**Authors:** Huihui Li, Xingyu Yan, Man Yang, Mei Liu, Shan Tian, Mengru Yu, Wei-Ping Li, Cong Zhang

**Affiliations:** ^1^Center for Reproductive Medicine, Ren Ji Hospital, School of Medicine, Shanghai Jiao Tong University, Shanghai 200135, China; ^2^Shanghai Key Laboratory for Assisted Reproduction and Reproductive Genetics, Shanghai 200135, China; ^3^Center for Reproductive Medicine, Department of Obstetrics and Gynecology, Qilu Hospital of Shandong University, Jinan 250012, China; ^4^Fujian Provincial Key Laboratory of Reproductive Health Research, Medical College of Xiamen University, Xiamen, Fujian 361102, China; ^5^Shandong Provincial Key Laboratory of Animal Resistance Biology, College of Life Sciences, Shandong Normal University, 88 East Wenhua Road, Jinan, Shandong 250014, China; ^6^Department of Obstetrics, Affiliated Hospital of Shandong University of Traditional Chinese Medicine, Jinan, Shandong 250011, China; ^7^Center for Reproductive Medicine, Shandong Provincial Hospital Affiliated to Shandong University, Shandong Provincial Key Laboratory of Reproductive Medicine, National Research Center for Assisted Reproductive Technology and Reproductive Genetics, The Key Laboratory for Reproductive Endocrinology of Ministry of Education, Jinan 250021, China; ^8^Center for Reproductive Medicine, Jinan Central Hospital, Jinan 250013, China

## Abstract

**Objective:**

Preeclampsia (PE) is a severe complication in pregnancy and a leading cause of maternal and infant mortality. However, the exact underlying etiology of PE remains unknown. Emerging evidence indicates that the cause of PE is associated with genetic factors. Therefore, the aim of this study is to identify susceptibility genes to PE.

**Materials and Methods:**

Human Exome BeadChip assays were conducted using 370 cases and 482 controls and 21 loci were discovered. A further independent set of 958 cases and 1007 controls were recruited for genotyping to determine whether the genes of interest *ROS1* and *PTPRK* are associated with PE. Immunohistochemistry was used for localization. Both qPCR and Western blotting were utilized to investigate the levels of PTPRK in placentas of 20 PE and 20 normal pregnancies.

**Results:**

The allele frequency of *PTPRK* rs3190930 differed significantly between PE and controls and was particularly significant in severe PE subgroup and early-onset PE subgroup. PTPRK is primarily localized in placental trophoblast cells. The mRNA and protein levels of PTPRK in PE were significantly higher than those in controls.

**Conclusion:**

These results suggest that PTPRK appears to be a previously unrecognized susceptibility gene for PE in Han Chinese women, and its expression is also associated with PE, while *ROS1* rs9489124 has no apparent correlation with PE risk.

## 1. Introduction

Preeclampsia (PE) is a severe complication in pregnancy and a leading cause of maternal and infant mortality; the worldwide PE incidence is 5–10% [1, 2]. PE is characterized by a new onset of hypertension and proteinuria after the 20th week of gestation. PE has been associated with abnormal differentiation and invasion of the trophoblast, widespread endothelial cell dysfunction, intravascular inflammation, impaired placental perfusion, oxidative stress, and angiogenesis imbalance [1]. However, the exact underlying etiology of PE remains unknown despite extensive efforts toward identifying the pathogenesis of PE.

Emerging evidence indicates that the cause of PE is associated with genetic factors since it was first expounded in the early 1960s [3, 4]. Pregnant mice lacking *catechol-O-methyltransferase, Corin*, *atrial natriuretic peptide*, or *Elabela* exhibit a PE-like phenotype [5–8]. In humans, geneticists [9] suggested that a single gene may possibly be responsible for PE. Besides, PE has also been shown to aggregate in families [9]. Studies have shown that the general heritability of PE is estimated to be about 55%, of which 35% is considered to be caused by maternal genetic factors, and 20% by fetal genetics [10]. Men born from preeclamptic pregnancy increase their partner's risk [11], which is perceived as a 13% contribution [10]. In addition, paternal obesity is the same as maternal obesity as a risk factor for PE [10]. Moreover, there is convincing evidence that the lead risk SNP rs4769613 from fetal *Fms-like tyrosine kin* (*FLT1*) is a risk factor for PE [12]. However, the present results remain controversial [13]. The genetic architecture behind PE is complex, as it includes maternal and fetal genes, genetically unfavorable combinations of maternal leukocyte receptors and fetal antigens, paternal factors, and any genetic conflict between the parents [11, 14, 15]. Therefore, a variety of candidate genes need to be investigated to identify the underlying causes of PE development.

To identify susceptibility genes, exome sequencing was conducted in a Han Chinese population, which identified several loci linked or associated with PE. Among these, SNP rs9489124 from c-ros oncogene 1 receptor tyrosine kinase (*ROS1*) and rs3190930 from protein tyrosine phosphatase receptor type K (*PTPRK*) were indicated as genetic risk factors for PE according to functional prediction. ROS1 is an orphan receptor tyrosine kinase of the insulin receptor family and a transmembrane protein with a typical tyrosine kinase sequence [16, 17]. *ROS1* has been confirmed as the gene of epithelial-mesenchymal interactions and has been shown to decrease the synthesis of extracellular matrix components in tissues [18]. Stimulation of the ROS1 receptor via phosphorylation has been associated with activated mitosis, morphological transformation, and epithelial cell migration [19, 20]. PTPRK is a member of the protein tyrosine kinase family and plays an important role in the regulation of the tyrosine dephosphorylation of intracellular substrates [21]. It has been reported to be involved in the regulation of cell survival, proliferation, adhesion, and migration of specific cell types both *in vitro* and *in vivo* [22, 23]. Furthermore, inactivation of PTPRK has been recently reported in several tumors [24], indicating that it can also act as a tumor suppressor by inhibiting cell cycle progression. However, the roles played by ROS1 and PTPRK in PE and their association with PE remain unknown to date.

Therefore, rs9489124 from *ROS1* and rs3190930 from *PTPRK* were further genotyped in additional independent samples of 958 PE patients and 1007 controls in our replication study. Moreover, the expression of *PTPRK* in the placentas of 20 PE and 20 normal pregnant women was also examined. The results indicate that *PTPRK* may be a susceptible candidate for PE.

## 2. Materials and Methods

### 2.1. Ethics Statement

The study was approved by the Ethics Committees of the First Affiliated Hospital of Nanjing Medical University, the Ethics Committees of the Second Affiliated Hospital of Nanjing Medical University, or the Ethics Committees of the Provincial Hospital Affiliated to Shandong University. Written informed consent to participate in this study was obtained from each patient.

### 2.2. Subjects

370 PE and 482 healthy controls were chosen for Human Exome BeadChip assays. A further 958 PE women and 1007 healthy pregnant women were recruited for genotyping. All samples were collected from the First Affiliated Hospital of Nanjing Medical University, the Second Affiliated Hospital of Nanjing Medical University, and the Provincial Hospital Affiliated to Shandong University from December 2009 to February 2015. The controls were randomly selected from contemporaneous normotensive women without antenatal medical or obstetric complications and who delivered a healthy neonate at term (>37 weeks of gestation). PE was defined as a new onset of gestational hypertension (the presence of blood pressure values ≥ 140/90 mm·Hg) and/or proteinuria (≥300 mg within a 24 h urine collection and/or urine dipstick protein ≥1+) after 20 weeks of gestation in previously normotensive and non-proteinuric women [25]. Severe PE (SPE) was defined as blood pressure >160/110 mmHg and/or 24 h urinary protein ≥5 g or urine dipstick protein ≥2+ [25]. Early-onset PE was defined as PE manifestation prior to the 34th gestational week and if it occurred thereafter, it was defined as late-onset PE. Exclusion criteria included major congenital anomalies, chronic hypertension, autoimmune disease, metabolic or cardiovascular disease, or renal disease. The placental tissues of normal pregnant women (*n* = 20) and PE patients (*n* = 20) were also obtained from the Provincial Hospital Affiliated to Shandong University.

### 2.3. Exome Sequencing and Quality Control (QC)

DNA samples of 370 cases and 482 healthy controls were extracted from peripheral blood using Wizard® genomic DNA Purification Kit (Promega, Madison, WI, USA) following the manufacturer's protocol. The chip detection was entrusted to Emei Tongde Technology Development Co., Ltd. Briefly, intact genomic DNA (>30 *μ*g/*μ*l) with A260/280 ≥ 1.8 was selected for Human Exome BeadChip assays (Illumina, San Diego, CA, USA). It contained 270241 SNPs that were used to uncover SNPs related to PE risk. The raw data of the chip scan was read using Illumina GenomeStudio software (V2011), and the preliminary screening was performed using the QC test to remove poor-quality SNP loci and individuals (i.e., pregnant women). Multivariable logistic regression analysis, adjusted for age, was performed to identify SNPs with *P* < 0.05 that were significantly considered to be associated with PE. The resulting SNPs were subjected to a further QC check to remove the following poor-quality variants: (1) call rate *<* 95%, (2) Hardy–Weinberg equilibrium *P* < 10^−3^, and (3) SNPs in sex chromosomes. Susceptibility genes were identified after data analysis, combining their functions. Subsequently, genotyping was performed in the replication study to further verify the candidate genes using enlarged cohorts.

### 2.4. Genotyping

Genomic DNA was extracted from peripheral blood samples using the QIAamp DNA mini kit (Qiagen, NY, USA) according to the manufacturer's protocol. Both SNPs (rs9489124 from *ROS1* and rs3190930 from *PTPRK*) were genotyped based on PCR assays in a thermal cycler (Bio-Rad Laboratories, USA). The PCR conditions were as follows: an initial denaturation step at 94°C for 5 min; 35 cycles of 30 s denaturation step at 95°C, 30 s annealing step at 58°C, and a 45 s extension step at 72°C; a 7 min final extension step was added at 72°C. The primer sequences were *ROS1* forward: 5′-ACT CTC CTT ACT GTT GC CCA CC-3′ and reverse: 5′-ATG GCT TTT TAC CTG GAT TTA ATT AG-3′; *PTPRK* forward: 5′-AAA GGG AGA AAA ATG CCA CGT-3′ and reverse: 5′-GAA ACC TGT CCA TCT ATT GAG CC-3′.

The PCR products were first analyzed via agarose gel electrophoresis and then sequenced using an automated sequencer (ABI PRISM 310; Applied Biosystems, CA, USA). The sequence variants were confirmed via three independent PCR runs, followed by sequencing in both directions.

### 2.5. Immunohistochemistry

Immunolocalization of PTPRK in human placental tissue was evaluated via indirect detection with the avidin: biotinylated-peroxidase complex kit (Vector Laboratories, Burlingame, CA, USA). In brief, paraffin-embedded tissue blocks were cut to 5 *μ*m thickness and mounted onto APES-coated slides. Antigen retrieval was performed using sodium citrate buffer solution (10 mmol/L, pH 6.5) prior to staining and endogenous peroxidase was quenched via 3% H_2_O_2_ for 20 min at room temperature. Nonspecific binding was blocked with horse serum albumin and then, the tissue sections were probed with a goat antibody against an internal region of human PTPRK (4 *μ*g/ml; cat no. sc30807, Santa Cruz Biotechnology, TX, USA) overnight at 4°C. The controls used pre-immune goat IgG for the primary antibody at the identical concentration. After three washes, tissue sections were incubated with a biotinylated universal antibody. The specific immunoreactivity was developed via 3-amino-9-ethylcarbazole (Vector Laboratories, Burlingame, CA, USA). Subsequently, the sections were counterstained with hematoxylin (Fisher Scientific, Pittsburg, PA, USA). Finally, the slides were mounted with neutral balsam, and then examined and digitally photographed using a BX53 F Olympus microscope (Olympus, Tokyo, Japan).

### 2.6. Western Blotting Analysis

Immunoblotting was conducted in accordance to the previously reported procedures [26–28]. 100 *μ*g protein extract of placental tissues was boiled at 95°C for 7 min, then separated on 10% SDS polyacrylamide gel electrophoresis (SDS-PAGE), and electrically transferred to a polyvinylidene difluoride membrane with routine procedures. Subsequently, nonspecific binding was blocked with 5% fat-free milk in Tris buffer for 60 min. The membranes were first incubated with primary antibody against PTPRK (1 : 1000 dilution, Santa Cruz, USA) overnight at 4°C, and then washed and incubated with horseradish peroxidase-conjugated secondary antibody. The protein was visualized using enhanced chemiluminescence (ECL) reagents from Amersham Biosciences (Piscataway, NJ, USA). All obtained values were normalized to the internal control (*β*-actin, 1 : 10000 dilution, Abcam, UK). Immunoreactive signals were analyzed via densitometry using NIH Image-J imaging analysis software (Bethesda, MD, USA).

### 2.7. RNA Extraction and qPCR Assays

The total RNA of placental tissues was extracted using an RNA extraction kit (Takara Inc., Kusatsu, Japan). The RNA concentration was determined using a Nanodrop 2000c spectrophotometer (Thermo Scientific, Waltham, MA, USA) [29]. cDNA was synthesized via reverse transcriptase M-MuLV (Promega Inc., Canada) using 1 *μ*g of total RNA and following the manufacturer's protocols. To quantify *PTPRK* mRNA levels in placental tissues, qPCR assays were conducted in 96-well plates using SYBR® Green PCR Master Mix (Applied Biosystems Inc., NY, USA). Cycling conditions were as follows: pre-denaturation at 95°C for 2 min, followed by 40 cycles of 95°C for 20 s, 60°C for 30 s, 72°C for 45 s, and 7 min at 72°C. All experiments were conducted in triplicate. Then, the levels of mRNA were normalized against those of *ACTB* and analyzed according to the 2^−ΔΔCt^ method. The sequence of *PTPRK* primers: forward: 5′-CTG CCT ACA ATG AAG GAG AAC G-3′ and reverse: 5′-AAT CTC TAC CCG TGA ATC CAG T-3′.

### 2.8. Statistical Analysis

The obtained numerical variables of clinical characteristics of PE patients and controls are expressed as means ± standard deviations (SD). Hardy–Weinberg equilibrium (HWE) tests were conducted using Haploview software (Broad Institute, Cambridge, MA, USA). Clinicopathological characteristics were compared via Student's *t*-test. The frequencies of alleles between PE cases and controls were compared via Chi-square test. Odds ratios (OR) and 95% confidence intervals (CIs) were calculated to assess the disease risk conferred by certain allele. Multiple hypothesis testing was carried out using the Benjamini–Hochberg method to control the false discovery rate (FDR) in unconditional logistic regression analysis. An FDR of 0.05 was exploited as a cut-off value to assess whether the obtained *P* values were significant [30]. Binary logistic regression was conducted to adjust potential confounding covariates (maternal age). Values of *P* < 0.05 are taken as statistically significant differences. Statistical analyses were conducted using SPSS statistical software (version 17.0; SPSS Inc., Chicago, IL, USA).

## 3. Results

### 3.1. Clinicopathological Characteristics

The clinical characteristics of PE cases and controls are listed in Table 1. Women with PE had a higher mean maternal age (*P* < 0.05), BMI (*P* < 0.05), systolic blood pressure (SBP) (*P* < 0.05), diastolic blood pressure (DBP) (*P* < 0.05), and low gestational age rate than controls (*P* < 0.05). Moreover, the maternal age, BMI, SBP, DBP, primiparous percentage, and low gestational age percentage in the SPE and early-onset PE groups were significantly higher compared with their counterparts except BMI in the early-onset PE group, whereas the gestational age at birth and the fetal birth weight were significantly lower in the PE group than those in the controls, while the primiparous percentage and fetal sex were not significantly different in the PE group compared to control group.

### 3.2. Exome Sequencing and Gene Screening

After preliminary screening, 263,039 SNP loci and 851 pregnant women qualified, including 482 cases of normal pregnant women and 369 patients with SPE. Then, logistic regression was used to investigate the relationship between each SNP and SPE. All SNPs were classified according to minor allele frequency (MAF), OR value, and the *P* value of the logistic regression. A total of 414 SNP sites were selected according to the criteria of 0.01 < MAF < 0.05 and *P* < 0.05. Illumina GenomeStudio software was used for further quality inspection of these sites to remove sites of poor quality and those that were not in the exon region (although this is the exon chip, there are still many sites in non-exon regions). The remaining 331 loci are OR > 1, *P* < 0.05, and OR < 1, *P* < 0.05. Further screening of these genes for functions in proliferation, migration, invasion, recessive liver and kidney injuries, and angiogenesis identified 21 promising susceptibility genes (Table 2). Among these, *ROS1* and *PTPRK* were selected for further study according to their functions. Thereafter, the rs9489124 from *ROS1* and rs3190930 from *PTPRK* were genotyped in the replication study, using another larger cohort of samples.

### 3.3. ROS1 rs9489124 and PTPRK rs3190930 Allele Frequencies in PE and Control Groups

The genetic polymorphism analyses of *ROS1* rs9489124 and *PTPRK* rs3190930 were performed for 958 PE patients and 1007 healthy controls. The genotype frequencies of both gene polymorphisms were all in Hardy–Weinberg equilibrium in both the PE and the control groups (*P* < 0.05), indicating a balance of population genetics data from the same Mendelian population.

The *PTPRK* rs3190930 allelic frequencies are presented in Table 3. The MAF differed significantly between the PE group and the control group (*P*=0.033, OR = 1.995, 95% CI = 1.104–3.605). Relative to the controls, an association with rs3190930 was observed in women with SPE (*P*=0.035, OR = 2.346, 95% CI = 1.166–4.722) but not in women with mild PE (*P*=0.120, OR = 1.763, 95% CI = 0.896–3.466). MAF also differed significantly between early-onset PE and controls (*P*=0.005, OR = 3.271, 95% CI = 1.521–7.037), while no difference was found between late-onset PE and controls (*P*=0.120, OR = 1.657, 95% CI = 0.871–3.151).

To further explore the relationship between *PTPRK* rs3190930 and PE, the investigated cases were divided into four subgroups: early-onset mild PE (76 cases), early-onset SPE (127 cases), late-onset mild PE (499 cases), and late-onset SPE (256 cases). The MAF differed significantly between early-onset SPE and control groups (*P*=0.004) (Table 4). No significant differences were found between the controls and other subgroups (Table 4). Additionally, as shown in Table 5, no significant differences were found for *ROS1* rs9489124 allele frequencies between PE and control groups despite age adjustment (*P* < 0.05).

### 3.4. PTPRK Expression Changes in PE and Normal Human Placentas

**Figure 1:**
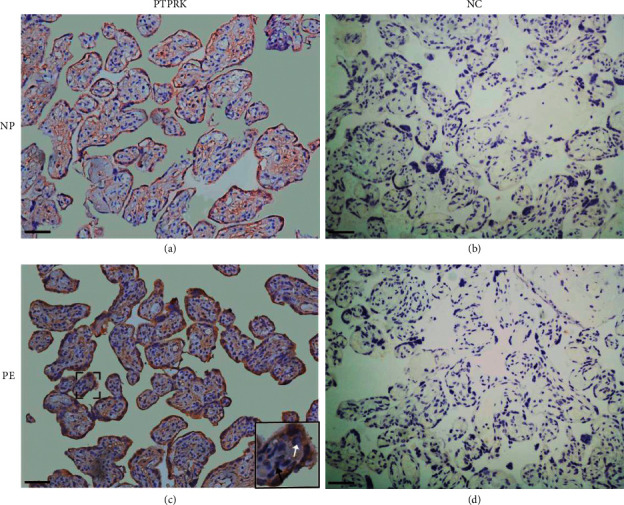
Immunolocalization of PTPRK in human placentas from normal pregnancies (NP) and preeclamptic (PE) pregnancies. (a, c) Brown staining of PTPRK. The tissue sections (*n* = 20 placentas for each group) were probed with PTPRK antibodies after hematoxylin counterstaining. (b, d) Negative control (NC). Preimmune IgG controls are shown in the right panel. The white arrow: syncytiotrophoblast. The black arrow: cytotrophoblast. Scale bars, 50 *μ*m.

**Figure 2:**
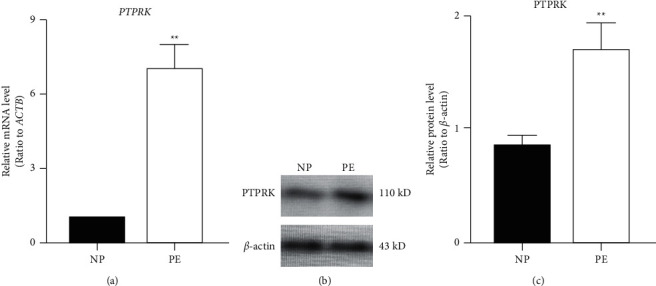
Expression patterns of PTPRK in human placentas. (a) QPCR analysis for *PTPRK* mRNA levels. The mRNA expression was determined using the 2^−ΔΔCt^ method with *ACTB* for normalization and was then compared to the control group (*n* = 20). (b, c) Western blotting analysis for the PTPRK protein levels (100 *μ*g of each placental sample) in human placentas from normal pregnancies (NP) and preeclamptic (PE) pregnancies. Representative blots are shown in (b), and the quantitative result is presented in (c). Data are expressed as mean ± SD of three independent experiments. ^*∗∗*^*P* < 0.01.

To determine the distribution of PTPRK in human placentas, immunohistochemistry was conducted. PTPRK was found to localize in normal pregnancies and PE placentas (Figure 1). In placental villi, PTPRK was primarily present in syncytiotrophoblasts and cytotrophoblasts (Figures 1(a) and 1(c)). Furthermore, the mRNA levels of *PTPRK* were measured in human placentas. As depicted in Figure 2(a), the *PTPRK* mRNA was expressed at higher levels in the PE group than that in controls. To quantify the changes in the PTPRK protein in human placentas, Western blotting analysis was conducted. PTPRK was detected at ∼140 kDa in human placental tissues (Figure 2(b)). When normalized to *β*-actin, the levels of the PTPRK protein were significantly increased (*P* < 0.01) in PE compared to controls (Figure 2(c)).

## 4. Discussion

The Human Genome Project has generated a wealth of data and contributes genetic information on common human disorders. Identifying the genetic contributions of complex diseases will advance diagnosis and therapy and will have far-reaching implications for public health [31]. The major tools of the Human Genome Project for the identification of disease susceptibility loci are the SNPs [31]. Whole exome sequencing is a powerful technique for the identification of novel genes of complex disorders [32]. Our recent exome sequencing identified 21 gene loci susceptible to PE and interestingly, two of these (*ROS1* and *PTPRK*) had completely opposite functions. Then, replication studies were conducted on rs9489124 from *ROS1* and rs3190930 from *PTPRK* to confirm their PE susceptibility in an independent and large cohort of Han Chinese women. Consequently, no variation in genetic background was found since all participants were Han Chinese women. A significant association between *PTPRK* rs3190930 and PE was identified. When the cases were divided into subgroups, statistically significant differences were also found between early-onset SPE and control groups. In contrast, no significant differences were observed between *ROS1* rs9489124 and the risk of PE even after age adjustment. In addition, our data demonstrated that more cases of early-onset PE were SPE compared to late-onset PE. Moreover, maternal age, BMI, and primiparity are risk factors for PE, especially for SPE and early-onset PE, which are consistent with previous studies [33–35].

PE is a complex disease during pregnancy. Hypoxia caused disruption of the angiogenic balance among vascular endothelial growth factor/placenta-derived growth factor and soluble Fms-like tyrosine kinase-1 (sFLT-1) is speculated to contribute to some of the maternal symptoms of PE [36–40]. Moreover, the absent or incomplete invasion of trophoblast cells into maternal spiral arteries has been suggested as initial steps in triggering PE [41]. Furthermore, it has been established that trophoblast invasion is influenced by a multitude of regulating factors such as adhesion molecules, cytokines, growth factors, proteases, and matrix-derived components [42–44]. Human *PTPRK* locates on the long arm of chromosome 6q22-23 and has been indicated as a tumor suppressor [21, 45]. Although the precise physiological role of PTPRK is still poorly understood, its ability to mediate homophilic or heterophilic interactions among cells together with the observation that the expression of PTPRK increases with increasing cell density, which strongly suggests that PTPRK plays a central role in the regulation of cell-cell contact formation [46]. In addition, it has been reported that the expression of E-cadherin, which is a classical cell-cell adhesion molecule, is increased in interstitial and vascular EVT cells in patients with PE [47, 48]. Its expression, however, is reduced from the first trimester to the third trimester in normal term placentas [49]. Therefore, it has been suggested that the loss of E-cadherin is likely playing a key role in the EVT cell invasion of maternal spiral arteries [50]. PTPRK has been previously shown to be associated with E-cadherin/*β*-catenin at the cell-cell contact area of adjacent cells, through dephosphorylation of E-cadherin/*β*-catenin [51, 52]. Reversible protein phosphorylation is a key regulatory mechanism in cell functions such as proliferation, differentiation, migration, and gene expression [53]. Thus, PTPRK appears to work as a molecular link between E-cadherin/*β*-catenin, which is involved in both cell adhesion and gene transcription [52]. The epidermal growth factor receptor (EGFR) is a major proliferative pathway and PTPRK specifically and directly dephosphorylates EGFR, thus acting as a major negative regulator of EGFR signaling. Therefore, our results that showed that PTPRK was significantly increased in PE patients are consistent with previous research, which demonstrates the upregulation of PTPRK in PE inhibited beta-catenin and EGFR tyrosine phosphorylation and the subsequent suppression of cell growth [52, 54], which might be part of the PE pathogenesis. However, the relationship and interaction between PTPRK and the tyrosine kinase sFLT, which is closely related to PE, need to be further studied.

ROS1 elicits its regulatory effects through the binding and phosphorylating of multiple factors, then activating diverse signaling cascades, which is central for cell proliferation, differentiation, and survival [55]. ROS1 can activate STAT3, which is essential for oncogenic receptor RTKs-dependent cell transformation [56]. It is also implicated in the stimulation of the PI3K/AKT signaling pathway, which is a canonical cascade that is responsible for both cell proliferation and growth [57]. Compelling evidence showed that *ROS1* can fuse with other genes, leading to protein kinase activation [58]. Several epithelial cancer types, including non-small-cell lung cancer, express activated fusion kinases that stimulate cancer progression [59]. These findings suggest that ROS1 and other proteins may act synergistically to mediate oncogenic function. However, *ROS1* rs9489124 is not related to PE even after age adjustment. This means that the conducted exome sequencing experiment has a false positive result and requires more samples for verification. Future studies should evaluate associations between PE and further genetic variants.

This study has some limitations. First of all, the function of PTPRK has not been studied in depth. Secondly, due to the case-control study design, our study is not enough to provide causality, but only an association study. However, our findings do help predict PE risk.

## 5. Conclusion

In conclusion, the new susceptibility gene *PTPRK* has been identified for PE in Han Chinese women; moreover, the expression levels of *PTPRK* are related to PE; that is, both the expression levels and the polymorphisms of *PTPRK* are associated with PE. However, *ROS1* SNP is not related to the risk of developing PE in the same population. The detailed pathogenic role of *PTPRK* in PE remains unclear and still requires functional studies in the future.

## Figures and Tables

**Table 1:** Clinicopathological characteristics of subjects (mean ± SD).

Characteristics	Control (*N* = 1007)	PE (*N* = 958)	PE		PE	
			MPE (*N* = 575)	SPE (*N* = 383)	Early-onset (*N* = 203)	Late-onset (*N* = 755)
Maternal age (year)	27.18 ± 4.43	28.83 ± 5.67^a^	28.12 ± 5.35	29.91 ± 5.97^b^	30.58 ± 5.76	28.36 ± 5.56^c^
BMI (before pregnancy)	21.93 ± 3.45	23.47 ± 3.78^a^	23.13 ± 3.83	24.00 ± 3.63^b^	23.70 ± 3.58	23.41 ± 3.83
SBP (mmHg)	117.40 ± 8.17	153.54 ± 57.76^a^	143.17 ± 7.48	169.11 ± 88.70^b^	167.47 ± 121.43	149.80 ± 14.74^c^
DBP (mmHg)	74.23 ± 6.88	100.36 ± 11.38^a^	94.46 ± 6.41	109.22 ± 11.48^b^	106.70 ± 13.80	98.66 ± 9.98^c^
Primiparous percentage	24.43	23.70	20.52	28.47^b^	34.98	20.66^c^
Gestational time (days)	277.29 ± 9.85	264.81 ± 17.99^a^	266.04 ± 16.57	262.82 ± 19.97^b^	256.55 ± 21.10	267.63 ± 15.88^c^
Low gestational age (percentage)	3.18	25.47^a^	18.09	36.55^b^	63.05	15.36^c^
Fetal weight (g)	3431.36 ± 1354.50	3037.20 ± 754.94^a^	3161.73 ± 677.95	2852.46 ± 823.51^b^	2281.09 ± 881.27	3217.23 ± 594.16^c^
Fetal sex (F/M)	466/541	492/466	265/310	201/182	110/93	356/399

Note: PE = preeclampsia; MPE = mild preeclampsia; SPE = severe preeclampsia; BMI = body mass index; SBP = blood pressure, systolic; DBP = blood pressure, diastolic. ^a^*P* value < 0.05 vs. control. ^b^*P* value < 0.05 vs. MPE. ^c^*P* value < 0.05 vs. early-onset PE.

**Table 2:** 21 susceptibility genes associated with PE in exome sequencing.

Gene	Gene ID	Chromosome	MAF	OR	*P*	Protein description
*COL16A1*	1307	1	0.0464	2.03	0.00340	Extracellular matrix
*COL11A2*	1302	6	0.0223	2.59	0.00691	Extracellular matrix
*COL6A2*	1292	21	0.0117	2.71	0.04289	Extracellular matrix
*SPARCL1*	8404	4	0.0135	3.58	0.00683	Extracellular matrix
*DOCK5*	80005	8	0.0182	3.03	0.00621	Guanyl-nucleotide exchange factor
*CYP2C9*	1559	10	0.0481	1.90	0.00695	Drug-metabolizing enzyme
***ROS1***	**6098**	**6**	**0.0334**	**1.84**	**0.02874**	**Tyrosine kinase activity**
*MAP4K5*	11183	14	0.0428	1.77	0.02371	Serine/threonine kinase activity
*AKAP9*	10142	7	0.0170	2.64	0.0157	Protein complex scaffold
*AKAP6*	9472	14	0.0105	2.82	0.03814	Ion channel and PKA binding
*AKAP11*	11215	13	0.0199	2.09	0.04979	Phosphatase 1 and PKA binding
*TRPM2*	7226	21	0.0258	2.10	0.02180	Transmembrane transporter activity
*ITPR3*	3710	6	0.0188	2.20	0.03567	Calcium release channel
*ITPRIPL1*	150771	2	0.0381	1.71	0.04394	ITPR3 interacting protein-like 1
*NSUN5*	55695	7	0.0288	3.13	0.00045	DNA methyltransferase
*WBSCR22*	114049	7	0.0152	3.72	0.00289	DNA methyltransferase
*NOTCH4*	4855	6	0.0199	2.70	0.00882	Notch signaling protein
*APCDD1L*	164284	20	0.0370	1.87	0.02093	Wnt signaling protein
*LGR5*	8549	12	0.0141	2.58	0.03131	Wnt signaling protein
*MAFF*	23764	22	0.0329	0.26	0.00014	Pitocin receptor- associated factor
***PTPRK***	**5796**	**6**	**0.0211**	**0.33**	**0.00710**	**Tyrosine phosphatase**

Note: MAF = minor allele frequency; OR = odds ratio.

**Table 3:** Allele frequencies of *PTPRK* rs3190930 in women with and without PE.

rs3190930		Allele (T/C)	MAF	*P*	*P*_adjust_	OR	*P*_adjust_^*a*^	OR_adjust_^a^
Control		17/1997	0.008					
PE		32/1884	0.017	0.020	0.033	1.995 (1.104–3.605)	0.003	2.363 (1.293–4.320)
PE	MPE	17/1133	0.015	0.096	0.120	1.763 (0.896–3.466)	0.074	1.871 (0.940–3.725)
	SPE	15/751	0.020	0.014	0.035	2.346 (1.166–4.722)	0.002	3.220 (1.552–6.683)
PE	Early-onset	11/395	0.027	0.001	0.005	3.271 (1.521–7.037)	<0.001	4.778 (2.108–10.833)
	Late-onset	21/1489	0.014	0.120	0.120	1.657 (0.871–3.151)	0.073	1.822 (0.947–3.506)

Note: PE = preeclampsia; MAF = minor allele frequency; OR = odds ratio between case and control groups; 95%CI = 95% confidence interval. The *P*_adjust_ value was obtained using the Benjamini–Hochberg method. *P*_adjust_^*a*^ value was adjusted by maternal age.

**Table 4:** Allele frequencies of *PTPRK* rs3190930 in control, early-onset mild PE, early-onset severe PE, late-onset mild PE, and late-onset severe PE subjects.

rs3190930	Allele (T/C)	MAF	*P*	*P*_adjust_	OR (95%CI)	*P*_adjust_^*a*^	OR_adjust_^a^
Control	17/1997	0.008					
Early-onset mild PE	3/149	0.020	0.160	0.320	2.365 (0.685–8.162)	0.122	2.780 (0.761–10.517)
Early-onset severe PE	8/246	0.031	0.001	0.004	3.820 (1.632–8.944)	<0.001	5.877 (2.312–14.938)
Late-onset mild PE	14/984	0.014	0.153	0.204	1.671 (0.820–3.404)	0.154	1.692 (0.820–3.489)
Late-onset severe PE	7/505	0.014	0.276	0.276	1.628 (0.672–3.948)	0.137	2.002 (0.801–5.001)

Note: PE = preeclampsia; MAF = minor allele frequency; OR = odds ratio between case and control groups; 95% CI = 95% confidence interval. The *P*_adjust_ value was obtained using the Benjamini–Hochberg method. ^a^The *P* value was adjusted by maternal age.

**Table 5:** Allele frequencies of *ROS1* rs9489124 in women with and without PE.

rs9489124		Allele (T/C)	MAF	*P*	OR	*P*_adjust_^*a*^	OR_adjust_^a^
Control		30/1984	0.015				
PE		39/1877	0.020	0.193	1.374 (0.850–2.221)	0.178	1.400 (0.856–2.311)
PE	MPE	24/1126	0.021	0.212	1.410 (0.820–2.423)	0.161	1.488 (0.854–2.592)
	SPE	15/751	0.020	0.382	1.321 (0.707–2.469)	0.390	1.335 (0.691–2.581)
PE	Early-onset	8/398	0.020	0.477	1.329 (0.605–2.921)	0.349	1.428 (0.646–3.443)
	Late-onset	31/1479	0.021	0.204	1.386 (0.835–2.300)	0.190	1.417 (0.841–2.385)

Note: PE = preeclampsia; MAF = minor allele frequency; OR = odds ratio between case and control groups; 95% CI = 95% confidence interval. ^a^The *P* value was adjusted by maternal age.

## Data Availability

The data used to support the findings of this study are available from the corresponding author upon request.

## References

[1] Mol B. W. J., Roberts C. T., Thangaratinam S., Magee L. A., de Groot C. J. M., Hofmeyr G. J. (2016). Pre-eclampsia. *The Lancet*.

[2] Osungbade K. O., Ige O. K. (2011). Public health perspectives of preeclampsia in developing countries: implication for health system strengthening. *Journal of Pregnancy*.

[3] Chesley L. C., Annitto J. E., Cosgrove R. A. (1968). The familial factor in toxemia of pregnancy. *Obstetrics & Gynecology*.

[4] Chesley L. C., Cosgrove R. A., Annitto J. E. (1962). Pregnancies in the sisters and daughters of eclamptic women. *Obstetrics & Gynecology*.

[5] Kanasaki K., Palmsten K., Sugimoto H. (2008). Deficiency in catechol-O-methyltransferase and 2-methoxyoestradiol is associated with pre-eclampsia. *Nature*.

[6] Ho L., van Dijk M., Chye S. T. J. (2017). ELABELA deficiency promotes preeclampsia and cardiovascular malformations in mice. *Science*.

[7] Werner F., Kojonazarov B., Gassner B. (2016). Endothelial actions of atrial natriuretic peptide prevent pulmonary hypertension in mice. *Basic Research in Cardiology*.

[8] Cui Y., Wang W., Dong N. (2012). Role of corin in trophoblast invasion and uterine spiral artery remodelling in pregnancy. *Nature*.

[9] Chesley L. C., Cooper D. W. (1986). Genetics of hypertension in pregnancy: possible single gene control of pre-eclampsia and eclampsia in the descendants of eclamptic women. *BJOG: An International Journal of Obstetrics and Gynaecology*.

[10] Cnattingius S., Reilly M., Pawitan Y., Lichtenstein P. (2004). Maternal and fetal genetic factors account for most of familial aggregation of preeclampsia: a population-based Swedish cohort study. *American Journal of Medical Genetics*.

[11] Galaviz-Hernandez C., Sosa-Macias M., Teran E. (2018). Paternal determinants in preeclampsia. *Frontiers in Physiology*.

[12] Gray K. J., Saxena R., Karumanchi S. A. (2018). Genetic predisposition to preeclampsia is conferred by fetal DNA variants near FLT1, a gene involved in the regulation of angiogenesis. *American Journal of Obstetrics and Gynecology*.

[13] Ugwumadu A. (2020). The jury is still out on the genetics of pre‐eclampsia. *BJOG: An International Journal of Obstetrics and Gynaecology*.

[14] Tong J., Niu Y., Chen Z. J., Zhang C. (2020). Comparison of the transcriptional profile in the decidua of early‐onset and late‐onset pre‐eclampsia. *Journal of Obstetrics and Gynaecology Research*.

[15] Dekker G. A. (2014). Pre-eclampsia - a disease of an individual couple. *Pregnancy Hypertension: An International Journal of Women’s Cardiovascular Health*.

[16] Lemmon M. A., Schlessinger J. (2010). Cell signaling by receptor tyrosine kinases. *Cell*.

[17] Roskoski R. (2017). ROS1 protein-tyrosine kinase inhibitors in the treatment of ROS1 fusion protein-driven non-small cell lung cancers. *Pharmacological Research*.

[18] Liu Z. Z., Wada J., Kumar A., Carone F. A., Takahashi M., Kanwar Y. S. (1996). Comparative role of phosphotyrosine kinase domains ofc-rosandc-retProtooncogenes in metanephric development with respect to growth factors and matrix morphogens. *Developmental Biology*.

[19] Sachs M., Weidner K. M., Brinkmann V. (1996). Motogenic and morphogenic activity of epithelial receptor tyrosine kinases. *Journal of Cell Biology*.

[20] Xiong Q., Chan J. L., Zong C. S., Wang L. H. (1996). Two chimeric receptors of epidermal growth factor receptor and c-Ros that differ in their transmembrane domains have opposite effects on cell growth. *Molecular and Cellular Biology*.

[21] Fischer E., Charbonneau H., Tonks N. (1991). Protein tyrosine phosphatases: a diverse family of intracellular and transmembrane enzymes. *Science*.

[22] Tonks N. K. (2006). Protein tyrosine phosphatases: from genes, to function, to disease. *Nature Reviews Molecular Cell Biology*.

[23] Liu M., Xie S., Zhou J. (2018). Use of animal models for the imaging and quantification of angiogenesis. *Experimental Animals*.

[24] Matsushita M., Mori Y., Uchiumi K. (2019). PTPRKsuppresses progression and chemo‐resistance of colon cancer cells via direct inhibition of pro‐oncogenicCD133. *FEBS Open Bio*.

[25] (2013). Hypertension in pregnancy. Report of the American college of obstetricians and gynecologists’ task force on hypertension in pregnancy. *Obstetrics & Gynecology*.

[26] Guo S., Yan X., Shi F., Ma K., Chen Z.-J., Zhang C. (2018). Expression and distribution of the zinc finger protein, SNAI3, in mouse ovaries and pre-implantation embryos. *Journal of Reproduction and Development*.

[27] Guo T., Zhang L., Cheng D. (2015). Low-density lipoprotein receptor affects the fertility of female mice. *Reproduction, Fertility and Development*.

[28] Cui L.-l., Yang G., Pan J., Zhang C. (2011). Tumor necrosis factor *α* knockout increases fertility of mice. *Theriogenology*.

[29] Wang N., Li H., Zhu Y., Li N., Chen Z.-J., Zhang C. (2020). Melatonin protects against Epirubicin-induced ovarian damage. *Journal of Reproduction and Development*.

[30] Wellcome Trust Case Control C. (2007). Genome-wide association study of 14,000 cases of seven common diseases and 3,000 shared controls. *Nature*.

[31] Newton-Cheh C., Hirschhorn J. N. (2005). Genetic association studies of complex traits: design and analysis issues. *Mutation Research: Fundamental and Molecular Mechanisms of Mutagenesis*.

[32] Sanders S. (2011). Whole-exome sequencing: a powerful technique for identifying novel genes of complex disorders. *Clinical Genetics*.

[33] Poon L. C., Shennan A., Hyett J. A. (2019). The International Federation of Gynecology and Obstetrics (FIGO) initiative on pre-eclampsia: a pragmatic guide for first-trimester screening and prevention. *International Journal of Obstetrics & Gynaecology*.

[34] Kabrhel C., Varraso R., Goldhaber S. Z., Rimm E. B., Camargo C. A. (2009). Prospective study of BMI and the risk of pulmonary embolism in women. *Obesity*.

[35] Pimentel C., Solene D., Frédérique J., Guillaume B., Jean L., Maëla L. (2019). What are the predictive factors for preeclampsia in oocyte recipients?. *Journal of Human Reproductive Sciences*.

[36] Vuorela P., Helske S., Hornig C., Alitalo K., Weich H., Halmesmäki E. (2000). Amniotic fluid-soluble vascular endothelial growth factor receptor-1 in preeclampsia. *Obstetrics & Gynecology*.

[37] Levine R. J., Maynard S. E., Qian C. (2004). Circulating angiogenic factors and the risk of preeclampsia. *New England Journal of Medicine*.

[38] Maynard S. E., Min J.-Y., Merchan J. (2003). Excess placental soluble fms-like tyrosine kinase 1 (sFlt1) may contribute to endothelial dysfunction, hypertension, and proteinuria in preeclampsia. *Journal of Clinical Investigation*.

[39] Sugimoto H., Hamano Y., Charytan D. (2003). Neutralization of circulating vascular endothelial growth factor (VEGF) by anti-VEGF antibodies and soluble VEGF receptor 1 (sFlt-1) induces proteinuria. *Journal of Biological Chemistry*.

[40] Krauss T., Pauer H. U., Augustin H. G. (2004). Prospective analysis of placenta growth factor (PlGF) concentrations in the plasma of women with normal pregnancy and pregnancies complicated by preeclampsia. *Hypertension in Pregnancy*.

[41] Redman C. W., Sargent I. L. (2005). Latest advances in understanding preeclampsia. *Science*.

[42] Lunghi L., Ferretti M. E., Medici S., Biondi C., Vesce F. (2007). Control of human trophoblast function. *Reproductive Biology and Endocrinology*.

[43] Knöfler M. (2010). Critical growth factors and signalling pathways controlling human trophoblast invasion. *International Journal of Developmental Biology*.

[44] Zhu Y. Q., Yan X. Y., Li H. (2021). Insights into the pathogenesis of preeclampsia based on the features of placentation and tumorigenesis. *Reproductive and Developmental Medicine*.

[45] Barghorn A., Speel E. J. M., Farspour B. (2001). Putative tumor suppressor loci at 6q22 and 6q23-q24 are involved in the malignant progression of sporadic endocrine pancreatic tumors. *American Journal Of Pathology*.

[46] Sap J., Jiang Y. P., Friedlander D., Grumet M., Schlessinger J. (1994). Receptor tyrosine phosphatase R-PTP-kappa mediates homophilic binding. *Molecular and Cellular Biology*.

[47] Brown L. M., Lacey H. A., Baker P. N., Crocker I. P. (2005). E-cadherin in the assessment of aberrant placental cytotrophoblast turnover in pregnancies complicated by pre-eclampsia. *Histochemistry and Cell Biology*.

[48] Li H. W., Cheung A. N. Y., Tsao S. W., Cheung A. L. M., O. W. S. (2003). Expression of e-cadherin and beta-catenin in trophoblastic tissue in normal and pathological pregnancies. *International Journal of Gynecological Pathology*.

[49] Floridon C., Nielsen O., Holund B. (2000). Localization of E-cadherin in villous, extravillous and vascular trophoblasts during intrauterine, ectopic and molar pregnancy. *Molecular Human Reproduction*.

[50] Shih I., Hsu M. Y., Oldt R. J., Herlyn M., Gearhart J. D, Kurman R. J (2002). The role of E-cadherin in the motility and invasion of implantation site intermediate trophoblast. *Placenta*.

[51] Fuchs M., Müller T., Lerch M. M., Ullrich A. (1996). Association of human protein-tyrosine phosphatase *κ* with members of the armadillo family. *Journal of Biological Chemistry*.

[52] Novellino L., De Filippo A., Deho P. (2008). PTPRK negatively regulates transcriptional activity of wild type and mutated oncogenic *β*-catenin and affects membrane distribution of *β*-catenin/E-cadherin complexes in cancer cells. *Cellular Signalling*.

[53] Hale A. J., Ter Steege E., den Hertog J. (2017). Recent advances in understanding the role of protein-tyrosine phosphatases in development and disease. *Developmental Biology*.

[54] Xu Y., Tan L.-J., Grachtchouk V., Voorhees J. J., Fisher G. J. (2005). Receptor-type protein-tyrosine phosphatase-*κ* regulates epidermal growth factor receptor function. *Journal of Biological Chemistry*.

[55] van der Geer P., Hunter T., Lindberg R. A. (1994). Receptor protein-tyrosine kinases and their signal transduction pathways. *Annual Review of Cell Biology*.

[56] Zong C. S., Zeng L., Jiang Y., Sadowski H. B., Wang L.-H. (1998). Stat3 plays an important role in oncogenic Ros- and insulin-like growth factor I receptor-induced Anchorage-independent growth. *Journal of Biological Chemistry*.

[57] Acquaviva J., Wong R., Charest A. (2009). The multifaceted roles of the receptor tyrosine kinase ROS in development and cancer. *Biochimica et Biophysica Acta (BBA)-Reviews on Cancer*.

[58] Uguen A., De Braekeleer M. (2016). ROS1 fusions in cancer: a review. *Future Oncology*.

[59] El-Deeb I. M., Yoo K. H., Lee S. H. (2011). ROS receptor tyrosine kinase: a new potential target for anticancer drugs. *Medicinal Research Reviews*.

